# Root-knot nematode infections and soil characteristics significantly affected microbial community composition and assembly of tobacco soil microbiota: a large-scale comparison in tobacco-growing areas

**DOI:** 10.3389/fmicb.2023.1282609

**Published:** 2023-12-01

**Authors:** Yi Cao, Ning Lu, Dongmei Yang, Minghe Mo, Ke-Qin Zhang, Caibin Li, Shenghua Shang

**Affiliations:** ^1^Guizhou Academy of Tobacco Science, Guiyang, Guizhou, China; ^2^State Key Laboratory for Conservation and Utilization of Bio-Resources in Yunnan, Yunnan University, Kunming, Yunnan, China; ^3^Bijie Tobacco Company of Guizhou Province, Bijie, Guizhou, China

**Keywords:** root-knot nematode, rhizosphere microorganisms, bacterial community, soil physical and chemical factors, large-scale geographical condition

## Abstract

**Introduction:**

Tobacco root-knot nematode (RKN) is a highly destructive soil-borne disease worldwide. However, there is a lack of research on the relationship between RKN and tobacco root microbial community composition under large-scale geographical conditions in China.

**Methods:**

In this study, we collected 65 samples from 28 main tobacco-growing areas across 10 provinces in China and conducted 16S rDNA sequencing to investigate the dynamic microbial changes in tobacco soil infected by RKN compared to healthy tobacco soil. Based on the analysis of rhizosphere soil bacterial communities, changes after RKN infection, and soil environmental factors.

**Results:**

We found the 28 tobacco-growing areas could be divided into two distinct groups with different microbial compositions and varying responses to RKN infection. In group1 of the provinces of Anhui, Henan, Shanxi, and Heilongjiang, Vicinamibacteria dominated the bacterial community, while Acidobacteriae was present in low abundance. In contrast, group2 of the other six provinces (Yunnan, Guizhou, Chongqing, Guangxi, Hubei, and Shandong) exhibited an opposite pattern. After infected by RKN, the genera *Chitinophaga* increased significant in group 1, while the genera *Rhodococcus* in group 2 exhibited a substantial increase. Alpha-diversity analysis revealed that RKN-infected tobacco exhibited a richer and more diverse rhizosphere soil bacterial community compared to healthy tobacco in most growing areas. A total of 12 kinds of soil environmental factors were measured in healthy and RKN-infected tobacco soil, and based on the co-occurrence and correlation analysis between environmental factors and microbial species, the pH level, calcium (Ca), magnesium (Mg), phosphorus (P), iron (Fe), and sodium (Na) were identified as key environmental factors influencing the population composition of rhizosphere microorganisms during RKN infection. We observed that RKN infection further increased the pH in weakly alkaline group 1 soil, while weakly acidic group 2 soil experienced a further decrease in pH. Furthermore, we identified three genera as potential biocontrol or plant growth-promoting bacteria for tobacco.

**Discussion:**

These findings provide valuable reference data for managing RKN disease in different tobacco-growing areas and contribute to the exploration of new and effective biological control methods.

## Introduction

Root-knot nematodes (RKN; *Meloidogyne* spp.) are soil-borne pathogens that can infect a wide range of crops and cause significant damage (Jeger et al., 2018; Hemmati and Saeedizadeh, 2020). Various plants can be susceptible to infection by RKN, such as tobaccos (Barker and Weeks, 1991), tomatoes (Liu et al., 2020; El-Ashry et al., 2021), cucumbers (Yin et al., 2021), peppers (Eldeeb et al., 2022), and eggplants (Sharma et al., 2021). These nematodes are highly pathogenic and can destroy host resistance. According to the Food and Agriculture Organization of the United Nations (FAO), plant parasitic nematode infection has been considered the fourth invasive type of disease to affect plants, with an annual loss of more than 157 billion dollars worldwide (Huang et al., 2020). In China, the problem of tobacco suffering from RKN disease and other soil-borne diseases has become increasingly severe, leading to yield losses ranging from 30 to 50% (Huang et al., 2020).

Over 100 RKN species have been reported worldwide (Jones et al., 2013), with *Meloidogyne incognita*, *M. hapla*, *M. arenaria*, and *M. javanica* being the major pathogens in China. Among them, *M. incognita* is the dominant species causing the most damage to tobacco (Cui et al., 2021). By penetrating the root tips of tobacco and moving to the vascular cylinder, RKNs can induce the formation of specialized feeding sites called “giant cells” (Kyndt et al., 2013; Bartlem et al., 2014; Escobar et al., 2015). As the proliferation of tissues surrounds the nematode feeding site, the root system is disrupted, and general metabolic functions such as the absorption and transportation of water and minerals are hindered, leading to wilting and withering (Cao et al., 2022). Moreover, RKN infection can also drive pathogen infections that may cause fusarium wilt, root rot, and bacterial diseases (Khan and Ahamad, 2020; Leonetti and Molinari, 2020).

To protect tobacco from soil-borne diseases such as RKN disease, farmers have adopted various practices, including crop rotation, soil fumigation, and pesticide application. However, these methods are only temporarily effective and have led to increasingly serious resistance. Scientists have conducted many studies to control RKN disease in tobacco, including identifying resistance genes to RKN (Filho et al., 2016; Banakar et al., 2020), developing efficient biocontrol agents against RKNs (Cao et al., 2022), studying interactions between plant-parasitic RKNs and their hosts (Walsh et al., 2017; Qin et al., 2022), and investigating microbial community changes in rhizosphere soil (Huang et al., 2020). Of all types of plant-parasitic nematode management strategies, biocontrol methods are considered safer and more practical options (Aioub et al., 2022). For example, co-fertilization with animal manure and rhizobacteria can suppress phytonematodes and enhance the production of cucumbers and tomatoes (Ali et al., 2022). The combination of abamectin and/or emamectin benzoate with *Purpureocillium lilacinum* and rhizobacteria has been proven to be effective against *Meloidogyne incognita* on tomatoes (El-Ashry et al., 2021).

The rhizosphere is a tiny area of 1–2 mm outside the root of plants (Philippot et al., 2013). It is an interface where plant–soil ecosystems frequently exchange energy and nutrients, and it contains a huge number of microorganisms (Ding et al., 2021). Some species may be beneficial to plant growth and health in rhizosphere soil, such as nitrogen-fixing bacteria, photosynthetic bacteria, and biocontrol microorganisms (Ding et al., 2021), while others may be harmful and lead to plant diseases, including *Rhizoctonia solani*, *Ralstonia solanacearum*, and *Fusarium oxysporum* (Navarrete et al., 2013). Plant root exudates serve as the primary food sources for microbes, which can “shape” a unique rhizosphere microbial community around plants and significantly impact the composition of the rhizosphere microbiome (Zhang et al., 2020; Liang et al., 2021). The “plant–soil-microorganism” ecosystem is a complex “holobiont.” Due to nematode infection, tobacco can regulate the plasticity of its root morphology and architecture in order to adapt to the new rhizosphere environment (Zeng et al., 2022). Simultaneously, this adaptation can modify the structure and function of soil microbial communities by affecting the availability of soil organic matter and nutrients (Wan et al., 2021). Numerous studies have demonstrated an intimate relationship between root-knot nematodes (RKN) and the diverse and abundant plant-associated microbiota. Several investigations have found that nematode infections often lead to an enrichment of beneficial bacteria for plants, which has been considered a plant-healing mechanism (Li et al., 2023). However, the most common consequence is complex diseases caused by bacterial pathogens resulting from microbial imbalances.

The main hypothesis of this study is that there may be specific patterns in the changes of rhizosphere microbial communities before and after RKN infection in different tobacco-growing areas in China. Moreover, certain microorganisms with biocontrol functions might play a role in resisting nematode infection. Our aim is to identify key factors that influence the occurrence of tobacco RKN disease by examining the relationship between RKN infections, bacterial communities, and soil environmental factors in various tobacco-growing regions. This research will be valuable in exploring new and effective biological control methods. To investigate this, we collected samples from 28 tobacco-growing areas across 10 provinces in China. We utilized 16S rDNA sequencing to explore microbial communities and conducted a detailed examination of the composition of rhizosphere bacteria in both healthy and infected plants.

## Materials and methods

### Sample collection

Soil samples were collected during the tobacco growing season, approximately 90–120 days after transplantation, from 28 major tobacco-growing areas in 10 provinces in China (namely, Yunnan, Guizhou, Chongqing, Guangxi, Anhui, Henan, Hubei, Shandong, Shanxi, and Heilongjiang). All tobacco farms used traditional cultivation methods, including seedling production, fertilization, pest control, and other field management practices. Soil samples were collected from healthy plants and plants infected with root-knot nematodes. In summary, five plots were randomly selected from each farm for field sample collection, and roots of 3–5 random plants were sampled from the middle of each plot. Finally, 15–25 roots of plants were collected and mixed in one sterile sampling bag. For each plant, a root segment of 5 cm length and 0.5–3 mm diameter was collected from near the base of the plant, along with any adhering soil particles. The root segments were mixed and placed in sterile sampling bags. One portion of the samples was frozen at −80°C for DNA extraction, while the other portion was used for soil physicochemical analysis. Detailed information for each sample is presented in Supplementary Table S1.

### DAN extraction, 16S rDNA amplification, and sequencing

Microbial DNA was extracted from the soil using the Power Soil DNA Isolation Kit (MoBio Laboratories, Carlsbad, CA, United States), following the standard protocol. The final concentration and purity of the genomic DNA were measured using NanoDrop 2000 (NanoDrop Technologies, Wilmington, DE, United States), and DNA quality was assessed by 1% agarose gel electrophoresis. Primers 27F (5′-AGAGTTTGATCCTGGCTCAG-3′) and 533R (5′-TTACCGCGGCTGCTGGCAC-3′) were used to amplify the V1-V3 variable region of the 16S rRNA gene. High-quality PCR products were extracted and purified from a 2% agarose gel. The purified PCR products were sequenced on the 454 GS FLX platform. Detailed sequence information for each sample is presented in Supplementary Table S2. All the sequenced data and the names of the accession numbers can be found at https://www.ncbi.nlm.nih.gov/bioproject/PRJNA971867.

### Bioinformatics and statistical analyses

The raw data were trimmed and filtered to obtain high-quality reads, which were clustered into operational taxonomic units (OTUs) with a similarity threshold of 97% using USEARCH v11.0.667 (Edgar, 2018) in the QIIME v1.7.0 pipeline (Caporaso et al., 2010). Taxonomic classification was assigned to representative sequences of each OTU using the QIIME pipeline based on the SILVA database (release 138). OTUs identified as chloroplasts or mitochondria were removed, and the filtered OTU table was normalized by the minimum number of reads across all samples. Chao1 and Shannon indices were calculated to measure alpha diversity and determine community richness and diversity. For beta diversity, Bray–Curtis distance metrics were calculated using QIIME. Principal coordinate analysis (PCoA) using Bray–Curtis distances was performed with the “Vegan” package in R v4.2.2 (Oksanen et al., 2019). Pearson correlation coefficients were computed using the “corrplot” R package. Co-occurrence networks were visualized using Gephi 0.9.5 software and Cytoscape 3.8.0.

### Determination of physical and chemical parameters of soil samples

A mixture of 10 g of soil with 50 mL of sterile water was prepared, and the pH was measured using a digital pH meter. The concentration of soil organic matter (OM) was determined by the potassium dichromate oxidation-sulfuric acid titration method using 5 g of dried soil (Jankauskas et al., 2006). Available phosphorus and potassium in the soil were measured using the methods described by Lin et al. (2021) and Bray (1945). Ammonium nitrogen (NH4^+^-N) was measured using a continuous flow analyzer (Tan et al., 2021). The concentrations of sodium (Na), zinc (Zn), iron (Fe), calcium (Ca), magnesium (Mg), manganese (Mn), and copper (Cu) were measured using a standard elemental analyzer, with methods referenced from Bao (2000). Detailed information on the chemical parameters of each sample is presented in Supplementary Table S3.

## Results

### The composition and structure of bacterial communities in the rhizosphere of 28 main tobacco-growing areas

We sequenced the 16S rDNA amplifications of 65 samples collected from 28 main tobacco-growing areas in 10 provinces in China. However, the library construction failed for three samples, namely, YN-5 from Yunnan province and SD-3 and SD-4 from Shandong province. In addition, three other samples (R56 from Henan province and R51 and R58 from Yunnan province) were removed from subsequent analysis due to insufficient sequence reads. As a result, we used 59 good quality samples for operational taxonomic unit annotation. These samples included 25 healthy samples, 24 diseased samples, and 10 samples from other plants. We identified 31,828 OTUs, which were assigned to 1,330 genera belonging to 47 phyla. Among all the bacterial OTUs, 18,101 were associated with healthy tobacco, assigned to 1,094 genera and 45 phyla, while 18,843 were associated with diseased tobacco, assigned to 1,099 genera and 42 phyla. Acidobacteriota was the dominant bacterial phylum in all soil samples from different areas, followed by Proteobacteria, Chloroflexi, Actinobacteriota, and Planctomycetota, accounting for approximately 80% and even more than 90% in some samples.

**Figure 1:**
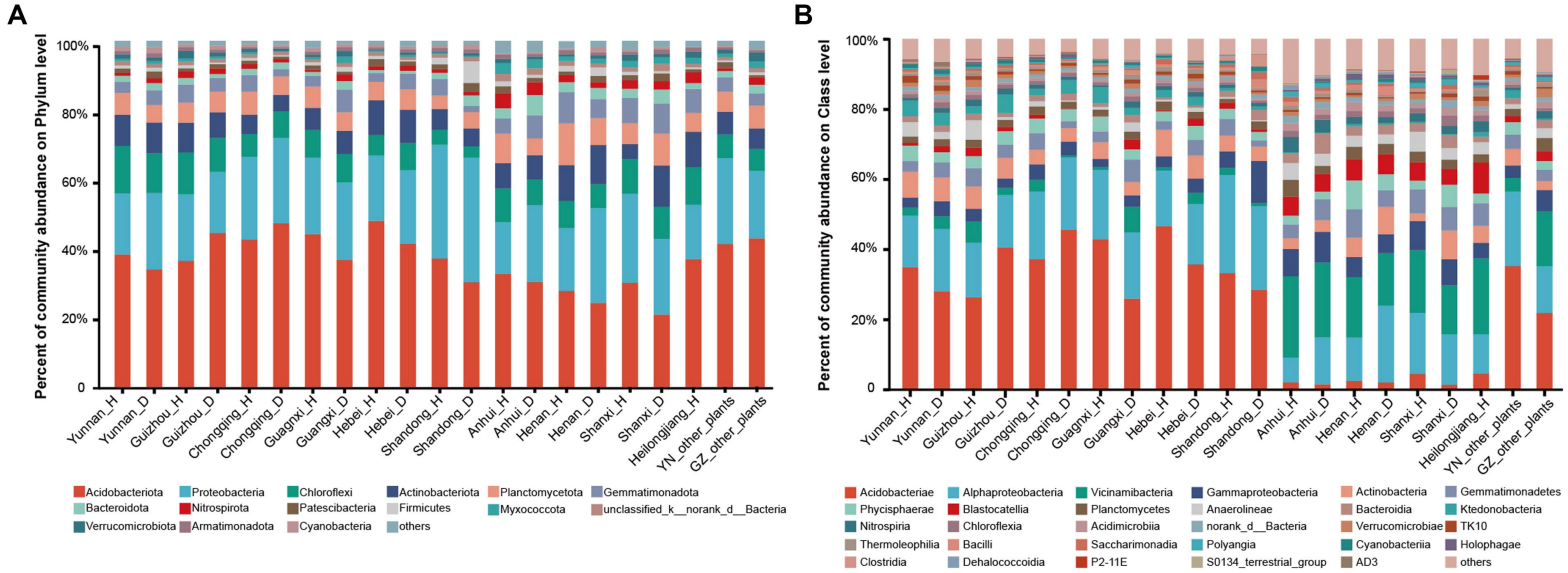
Composition of microorganisms in healthy and RKN-infected tobacco from different provinces. **(A)** Composition of microorganisms at the phylum level. **(B)** Composition of microorganisms at the class level.

Composition of microbial communities in the rhizosphere of samples collected from other plants (healthy forest, maize, vegetable, and fruit) in Yunnan and Guizhou provinces was almost the same as those from tobacco plants at the phylum, class, and genus levels (Figure 1; Supplementary Figure S1). The rhizosphere bacterial communities of healthy tobacco and RKN-infected tobacco were similar, especially in terms of the dominant taxa across all samples. However, there were differences between the percentage of exact species and the composition of some endemic species, most of which accounted for a low percentage. Detailed information is presented in Supplementary Table S4.

Based on the analysis of microbial communities, we discovered distinct microbial composition patterns at the class level between samples collected from Anhui, Henan, Shanxi, and Heilongjiang provinces and samples collected from the other six provinces (Yunnan, Guizhou, Chongqing, Guangxi, Hubei, and Shandong). Samples collected from the four former provinces tended to be dominated by bacteria in the Alphaproteobacteria and Vicinamibacteria taxa, followed by the Gammaproteobacteria and Actinobacteria classes, while the class Acidobacteria accounted for a low percentage, even less than 2%. In contrast, the latter six provinces exhibited a diametrically opposed situation. For instance, the taxon Vicinamibacteria was a minority class, and the class Acidobacteria predominated the community (more than 40%, Supplementary Table S4) of soil samples from these provinces (Figure 1B). To further characterize these patterns, we assigned the four provinces to group 1 and categorized the remaining six provinces as group 2.

### RKN infection drove the establishment of rhizosphere bacterial communities

The Venn diagram revealed that 884 genera, classified into 38 phyla, were shared by both healthy and RKN-infected tobacco, while 197 unique genera belonging to eight phyla were only found in the soil of healthy tobacco. On the other hand, 187 unique genera, all belonging to the phylum Halanaerobiaeota, were only found after RKN infection (Supplementary Table S5). This finding provides a clue for further research on biomarkers in microbial community diversity induced by RKN infection.

**Figure 2:**
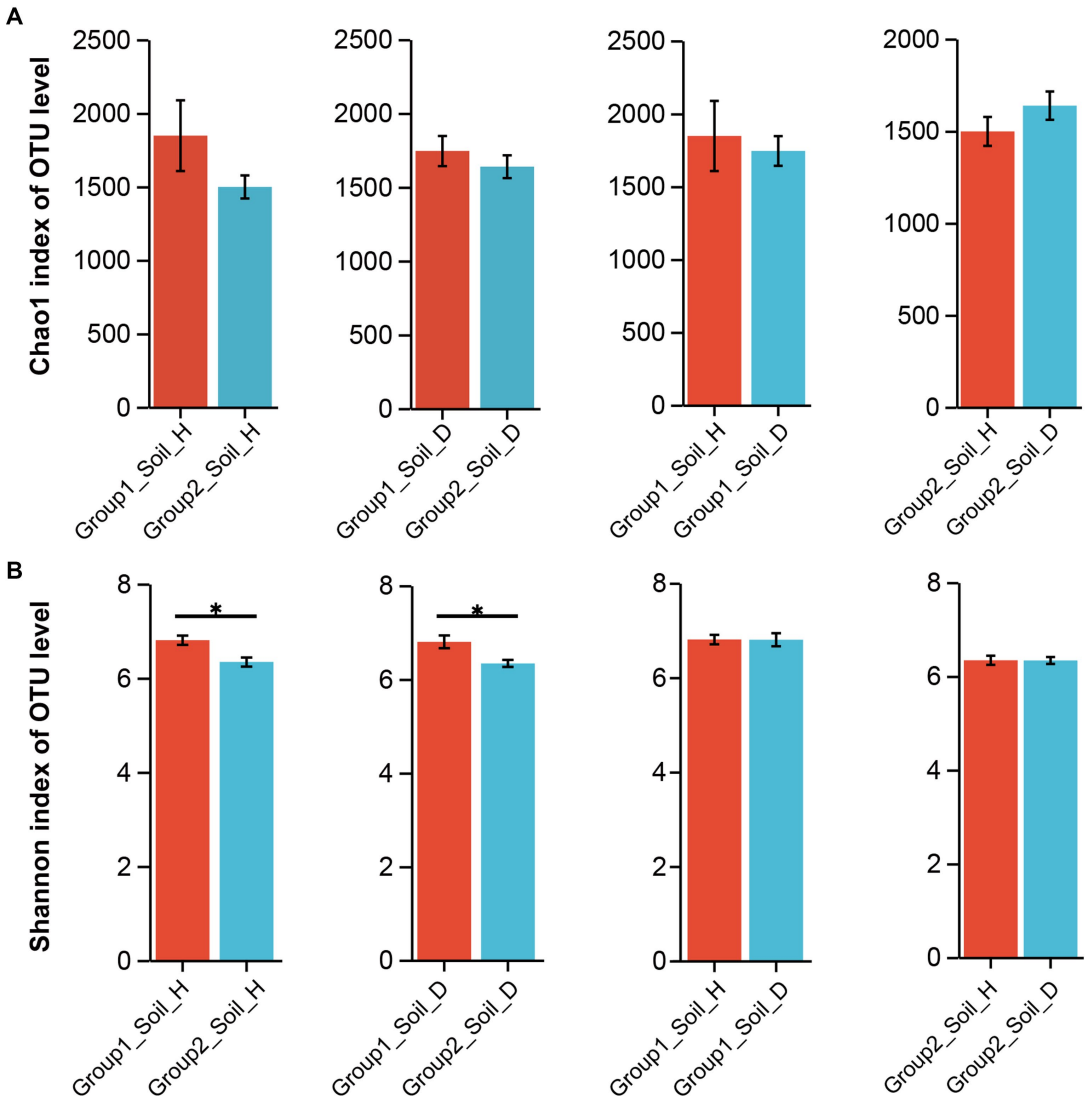
Changes in richness and diversity of rhizosphere soil microorganisms of healthy and RKN-infected tobacco on OTU level. **(A)** Statistics of microorganisms between group1 and group2 rhizosphere soil based on Chao1 index. **(B)** Statistics of microorganisms between group1 and group2 rhizosphere soil based on Shannon index.

Alpha diversity analysis was conducted to examine the species diversity between and among samples from different tobacco-growing provinces. No significant differences were observed within each province before and after RKN infection at the phylum, genus, and OTU levels (data not shown). However, when comparing group 1 samples (from the four provinces) with group 2 samples (from the other six provinces), we found that the rhizosphere soil of group 1 samples exhibited higher richness and diversity of bacteria based on the Chao1 index and the Shannon index results (Figure 2). Although there was no statistically significant difference between the rhizosphere soil microbiota of healthy tobacco and nematode-parasitized tobacco in both group 1 and group 2 samples, RKN-infected tobacco tended to exhibit greater richness and diversity of bacterial species in the rhizosphere soil compared with healthy tobacco.

It is worth noting that the rhizosphere soil of diseased tobacco had greater bacterial richness but lower evenness. For example, samples from Guizhou province (Supplementary Figure S2) indicated that disease occurrence led to the recruitment of more types of microbes with low abundance around the roots, resulting in a decline in overall evenness and a subsequent reduction in the value of the Shannon index. In contrast to the samples from other regions, samples from Chongqing exhibited an opposing result, where the rhizosphere soil of healthy tobacco had higher bacterial richness and greater bacterial diversity compared with the diseased samples (Supplementary Figure S2).

### Comparison of root microbial composition after RKN infection in different tobacco-growing areas

**Figure 3:**
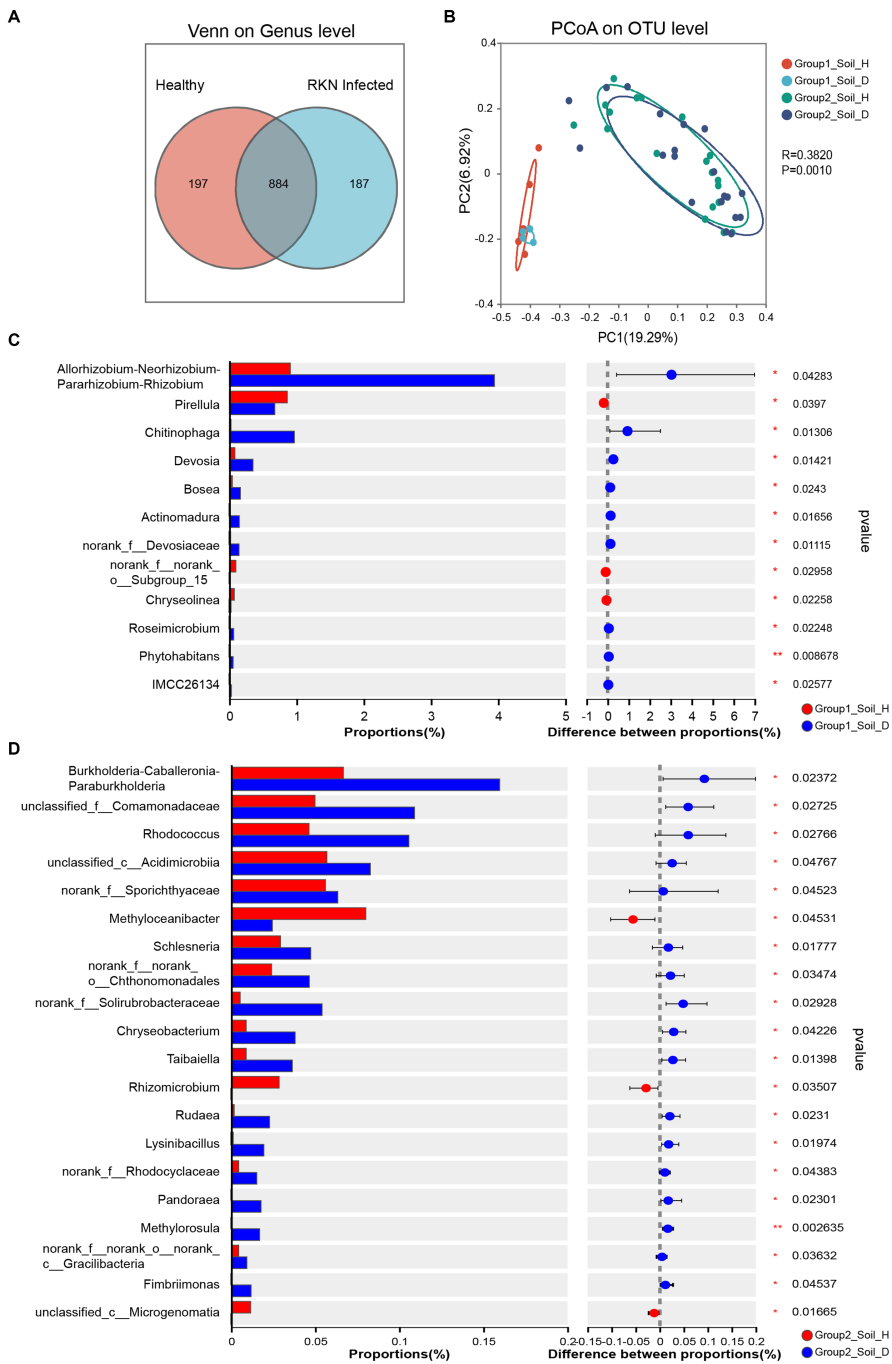
Community diversity measurements of microbial species in RKN-infected and healthy tobacco root rhizosphere. **(A)** Venn diagram showing the shared and unique genera between healthy and RKN-infected tobacco root soil. **(B)** PCoA analysis of bacterial communities in the rhizosphere based on Bray-Curtis distances at the OTU level among healthy and RKN-infected tobacco root soil in group1 and group2. **(C, D)** Enrichment of specific taxa at genus level in the rhizosphere of healthy tobacco plants against RKN-parasitized samples. Two-way ANOVA was conduced to determine the enriched species in healthy and infected plants (*p < 0.05, **p < 0.01, ***p < 0.001).

Principal coordinate analysis (PCoA) of Bary–Curtis distances revealed that all samples could be significantly separated into two groups, closely corresponding to group 1 and group 2 (Figure 3B). The PC1 component, which contributed the most to the differentiation, explained 19.29% of the total variance. This result indicated a significant differentiation of bacterial communities across latitudes (all provinces in group 1 are located north of the Yangtze River, while provinces in group 2 except Shandong are located south of the Yangtze River).

Further comparisons of changed microorganisms between healthy and infected tobacco showed significant differences between group 1 and group 2. RKN infection resulted in a marked change in bacterial abundance, with nine genera in group 1 and 17 genera in group 2, showing a sharp increase after infection, while only three genera declined (Figures 3C,D). Interestingly, none of the significantly changed bacteria belonged to the same genus. After infected by RKN, genera *Allorhizobium-Neorhizobium-Pararhizobium-Rhizobium* and *Chitinophaga* increased by 4.35 and 38.16 times, respectively, in group 1, while the genera *Burkholderia-Caballeronia-Paraburkholderia*, *Rhodococcus*, and *norank_f__Solirubrobacteraceae* in group 2 exhibited a substantial increase of more than 2- and even 10-fold in some cases. However, the genus *Chryseolinea* in group 1 and genus *Rhizomicrobium* in group 2 decreased to a very low level. Notably, the proportion of all significantly changed bacteria in the abundance was very low, indicating that they may play a critical role as a keystone species in RKN infection or defending against further damage to tobacco.

### Probiotic flora suggesting potential biocontrol bacteria

Biological control is considered a very promising approach for preventing or weakening RKN infection and other microbial pathogens. 16S rRNA gene sequencing is considered to be an effective approach to identify microorganisms with biocontrol or growth-promoting properties. In our study, based on the 197 unique genera found in healthy tobacco, we conducted additional analyses to identify specific types of bacteria, ultimately selecting three genera as potential probiotic candidates. The genus *Rhizomicrobium* primarily exists in the rhizosphere soil of plants, forming a symbiotic relationship with plant roots and exerting a series of beneficial effects on plant growth and health. For example, *Rhizomicrobium* sp. can produce substances such as plant hormones and trace elements, thereby facilitating plant growth and development. It also possesses the ability to inhibit the growth of plant pathogens, aiding plants in defending against diseases. The gram-negative genus *Dinghuibacter* exhibits diverse metabolic abilities and ecological functions, playing a significant role in carbon cycling and organic matter degradation. Finally, the genus *Chitinimonas* is considered to have the ability to degrade chitin, providing essential nutrients and energy for other microbial communities and promoting the health and balance of plant rhizosphere microbiota.

### Multiple soil environmental factors significantly correlated with RKN disease

**Figure 4:**
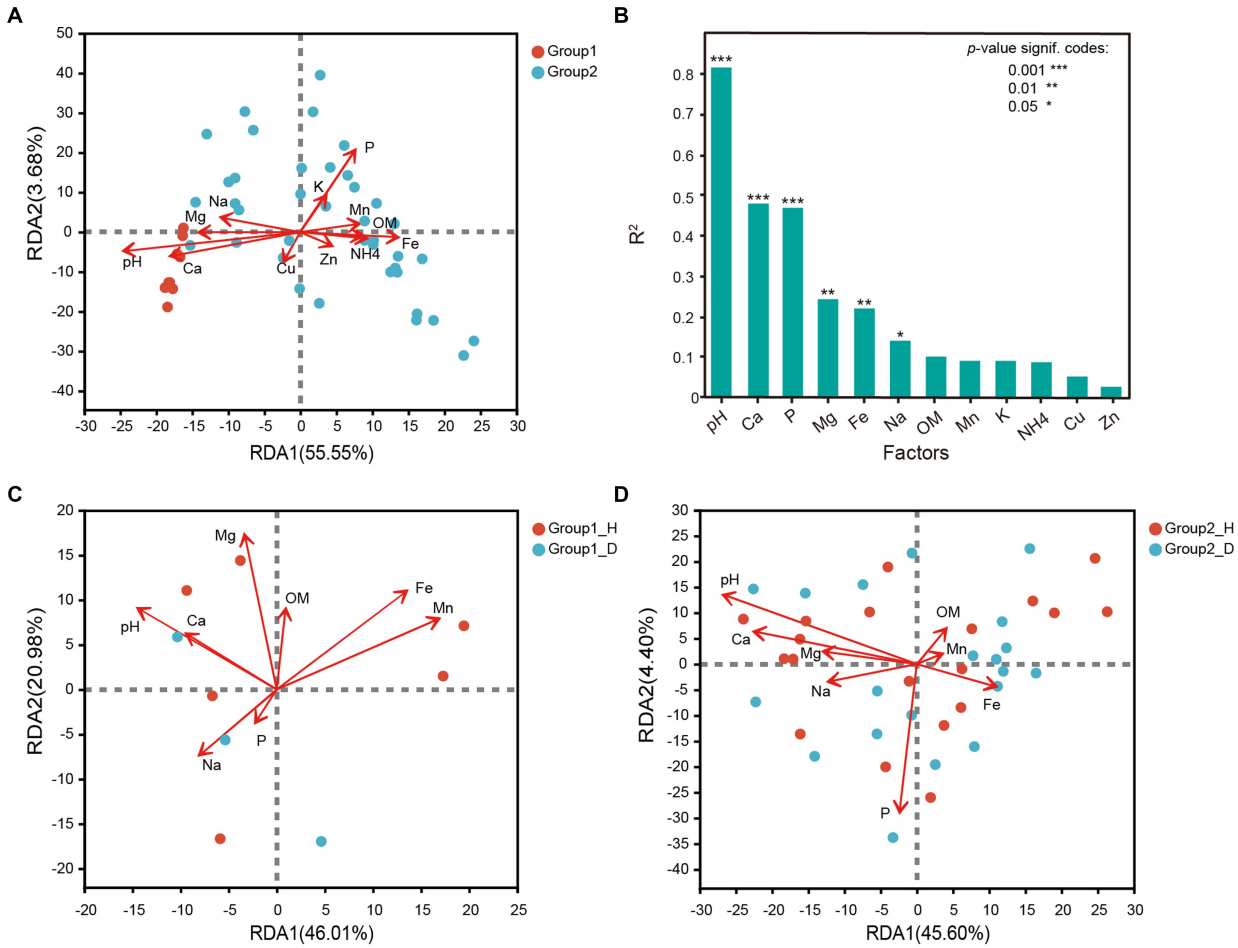
Correlation analysis between the bacterial community structure at genus level and soil environmental factors. **(A)** Redundancy analysis of soil environmental factors and all samples in group1 and group2. **(B)** Correlation analysis between chemical parameters of soil and the microbial community composition. **(C,D)** Redundancy analysis of soil environmental factors and samples with and without RKN disease in group1 and group2.

In this study, 12 types of soil environmental factors were measured, and the detailed results are presented in Supplementary Table S3. Based on variance inflation factor (VIF) selection and redundancy analysis, all the 12 chemical parameters exhibited some degree of correlation with the bacterial community structures at the OTU level. Redundancy analysis revealed that the bacterial community in group 1 and group 2 responded differently to these factors. Group 1 had weakly alkaline soil with higher calcium (Ca) and magnesium (Mg) contents, while group 2 had weakly acidic soil with lower phosphorus (P) and iron (Fe) contents. Soil pH and the metals Ca, Mg, and Na were identified as key parameters significantly correlated with rhizosphere microbial communities of tobacco root. On the other hand, P, Fe, and manganese (Mn) significantly but negatively affected bacterial community structures. Zinc (Zn), copper (Cu), potassium (K), and ammonium-N (NH4^+^-N) were considered insignificant as they had less impact on microbial distribution (Figure 4B). Further analysis revealed that, Mn (*R*^2^ = 0.5087, *p* = 0.020) was the most important effector affecting microbial distribution between healthy and RKN-infected tobacco in group 1, followed by Mg (*R*^2^ = 0.4543), Fe (*R*^2^ = 0.444), and pH (*R*^2^ = 0.424; Figure 4C). In group 2, pH (*R*^2^ = 0.6931, *p* = 0.001) had a significant influence on microbial distribution, while P (*R*^2^ = 0.5549, *p* = 0.001) and Ca (*R*^2^ = 0.4047, *p* = 0.001) were also key parameters (Figure 4D). Both in group 1 and group 2, pH and Ca had a consistent impact on bacterial distribution and had no correlation with parameter P. Interestingly, pH was found to change due to RKN infection, with an increase in alkaline soil and a decrease in acidic soil.

### Co-occurrence networks for bacterial communities between healthy and RKN-infected plants

**Figure 5:**
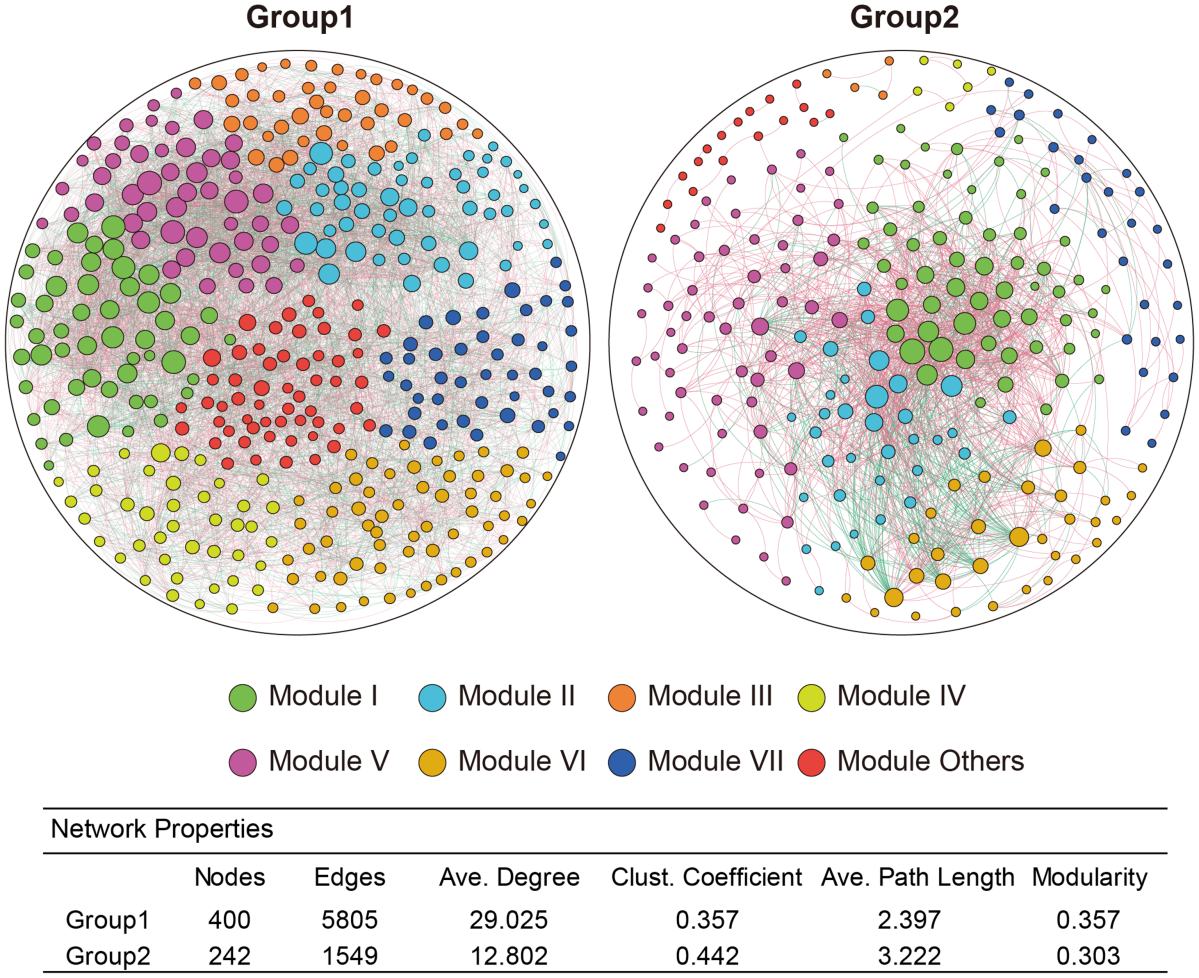
Networks of rhizosphere communities in soils of group1 and group2. The top 400 genera in abundance were selected for co-occurrence and correlation analysis. Genera with an absolute correlation value greater than 0.6 were selected for further analysis. Every node represents a different genus, and a large size of the node means a more complex correlation with other genera. Colours of each node represents eight modules with different co-occurrence. Edge with red colour means a positive correlation while green colour means a negative correlation. Thicker connections indicate higher correlation between genera.

To investigate microbial co-occurrence and correlation between environmental factors and microbial species in soil bacteria from both healthy and RKN-infected tobacco plants, the study selected the top 400 genera in abundance from both group 1 and group 2 for further analysis. The network results showed that while there were a large number of bacteria sharing complex interaction relationships in group 1 between healthy plants and RKN-infected plants, there were few genera sharing interactions in group 2. Based on these interactions, all the taxa could be divided into more than eight different modules (Figure 5), with varying species and different connections. The network complexity could be evaluated by the number of nodes, edges, and average degrees in each group. Taken together, there was a more complex bacterial interaction in group 1 (up to 400 genera over 33 phyla) than in group 2 (only 242 genera from 19 phyla with interactions). Certain genera, such as *Ferruginibacter, Ramlibacter, Sumerlaea, Kribbella, norank_f__norank_o__norank_c__OM190*, *norank_f__norank_o__11–24*, *unclassified_c__Gammaproteobacteria*, *norank_f__norank_o__norank_c__Subgroup_5*, *unclassified_o__Acidobacteriales,* and *norank_f__norank_o__SBR1031* tended to be more important as they had significantly positive or negative correlations with more than 70 types of other genera based on the changes in soil bacterial community structures of healthy and RKN-infected plants of group 1. In contrast, the top 10 genera having interactions with other bacteria in group 2 were *norank_f__Vicinamibacteraceae*, *UTCFX1*, *norank_f__A4b, norank_f__Roseiflexaceae*, *norank_f__norank_o__Vicinamibacterales*, *Pirellula*, *Ilumatobacter, Nordella*, *Steroidobacter,* and *Acidothermus*, and none of them were the same in group 1. Many genera were associated with different species, and their interactions with each other may be diverse. For instance, *Ferruginibacter* had a strong positive correlation with *Streptomyces*, *Ellin6055,* and *Kribbella* but negatively related to *Haliangium*, *Paludibaculum,* and *Nitrospira* in group 1 (Supplementary Table S7, Sheet 1), whereas in group 2, *Pirellula* was directly proportional to *UTCFX1* and *Tundrisphaera* and inversely correlated with *Acidothermus* and *Actinospica* (Supplementary Table S7, Sheet 2).

**Figure 6:**
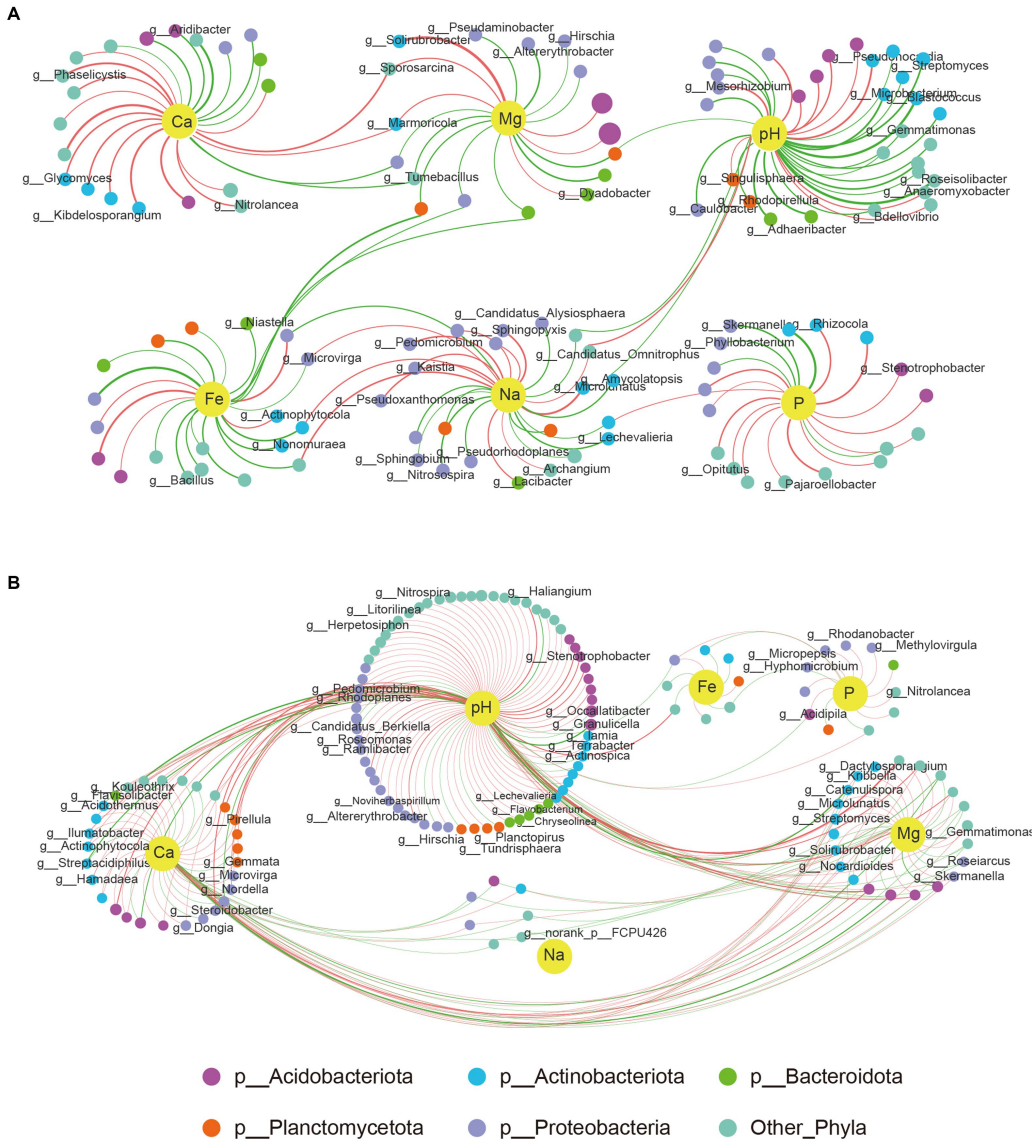
Networks of genera and chemical parameters of soil. **(A)** Correlations between genera with six soil environmental factors in group1. **(B)** Correlations between genera with six soil environmental factors in group 2. A big size of each node means a higher abundance at genus level. Colors of each genus represents six phyla. Edge with red color means a positive correlation while green color means a negative correlation. Thicker connections indicate higher correlation.

Our study also investigated the impact of six soil environmental factors (pH, Ca, P, Mg, Na, and Fe) on changes in microbial communities. The results showed distinct differences between group1 and group2 (Figure 6A). In group 1, every soil factor showed relationships with some exact species, and only a few species were affected by up to two environmental factors. However, there was a complex correlation between environmental factors and bacterial structures in group2. pH was the most critical factor (correlating with up to 119 genera), followed by Ca (correlating with 57 genera) and Mg (correlating with 30 genera). Species were significantly affected by Mg and also affected by pH and Ca at the same time. None of the genera except the unknown *g__norank_p__FCPU426* had a correlation with Na (Figure 6B). Five genera from the phylum Proteobacteria, three genera from phyla Bacteroidota, Acidobacteriota, and Planctomycetota, and another three genera belonging to other phyla showed high correlation with P, while only eight genera from phyla Planctomycetota, Actinobacteriota, and Acidobacteriota significantly correlated with Fe (Figure 6B).

Some genera, such as *Solirubrobacter*, were mainly positively correlated with Mg with a high level in group 1, whereas in group 2, not only Mg but also pH and Ca had a positive correlation with it, despite a low correlation degree. Other two genera, namely, *Sporosarcina* and *Marmoricola*, shared a significant correlation with Mg and Ca in group 1 but not with group 2. This phenomenon is not uncommon in other species–environment correlations, suggesting that differences in the composition of flora between group 1 and group 2 may account for the varying correlations between bacterial communities and environmental factors.

## Discussion

### Tobacco-growing areas can be divided into two groups based on key indicator differences

Based on the 16S rDNA sequencing results, the rhizosphere bacterial communities of different areas could be divided into two groups: group 1 (Anhui, Henan, Shanxi, and Heilongjiang) in the north and group 2 (Yunnan, Guizhou, Chongqing, Guangxi, Hubei, and Shandong) in the south. Notably, the Shandong Tobacco Farm, located in North China, had a highly similar rhizosphere microflora composition to those in South China and Southwest China. While there were large differences in the community structure and taxa composition at the genus and class levels, there was a high degree of consistency at the phylum level between group1 and group 2 (Figure 1; Supplementary Figure S1). Further analysis revealed that group 1 had weakly alkaline soil with higher Ca and Mg content, while group 2 had weakly acidic soil with higher P and Fe content. These differences in pH and nutrient levels likely account for the variation in flora between the two groups. Calcium and magnesium ions can react with water molecules to form hydroxide ions, thereby increasing the pH of the soil (Weil and Brady, 2017), while phosphorus may contribute to soil acidification by forming phosphate (Wang P. et al., 2022). In southern China, iron (Fe) often forms salts, leading to increased hydrogen ion concentration in the soil and subsequent pH decrease. In southern China, iron (Fe) often forms salts and promotes the increase of hydrogen ion concentration in soil, thus leading to the decrease in pH (Yan et al., 2020). Taken together, the difference of pH may be one of the major reasons for the variation in flora between the two groups.

### Changes in species richness and composition of microorganisms after RKN infection

The alpha diversity results showed that the richness and diversity of microbial flora in weakly alkaline group 1 soil were higher than those in weakly acidic group 2 soil, irrespective of the presence of RKN infection (Figure 2). This suggests that acidic soil may inhibit the growth of many microbial species. Although there was no significant change in microbial community diversity after RKN infection in both group 1 and group 2, there was a significant change in species richness and composition of microorganisms, which was similar to our previous study (Cao et al., 2022). After infection, approximately 20% of the total genera in healthy and diseased tobacco plants exhibited unique rhizosphere bacterial composition (Figure 3A). Additionally, the species richness decreased in group 1 but increased in group 2 after infection (Figure 2A), although Chongqing showed a contrary trend (Supplementary Figure S2). Previous studies indicated that the RKN infection may lead to an increase in the diversity of microbial flora, which may be caused by changes in plant root phenotypic traits (Zeng et al., 2022) and increased root secretions such as organic acids to recruit more microbial flora with beneficial effect on plants (Wang H. et al., 2022). Another hypothesis suggests that parasitizing nematodes may introduce their own microbial flora to the root site, resulting in changes in rhizosphere bacterial population diversity (Li et al., 2023). However, based on our extensive research spanning different tobacco-growing areas, we found that the influence of RNK infection and the changes in rhizosphere microflora had various characteristics. This comprehensive result may be attributed to a variety of factors, including the local climate, tobacco varieties, and the interactions between soil and other environmental factors.

### Impact of RKN infection on rhizosphere microbial community

Root-knot nematode infection resulted in changes in the relative abundance of rhizosphere microorganisms. For example, after RKN infection, there was a significant increase in the phylum Bacteroidota in group 1 and a minimal increase in group 2. In both group 1 and group 2, more taxa were observed, such as phyla Proteobacteria, Actinobacteriota (Figure 1A), Patescibacteria, Firmicutes, and Cyanobacteria. Bacteroidota is known to play a crucial role in soil carbon and nitrogen cycle, plant–microbial interaction, and humus degradation (Larsbrink and McKee, 2020). Proteobacteria and Actinobacteriota, which are highly abundant in plant rhizospheres (Figure 1A), are essential for maintaining rhizosphere health, as reported in previous studies (Cao et al., 2022; Wang H. et al., 2022). However, the function of Patescibacteria remains unclear due to its challenging isolation and limited research. Acidobacteriota and Nitrospirota exhibited higher relative abundance in group 1 of healthy plant soil but increased in group 2 after infection. Acidobacteriota could produce hydrogen ions (H^+^) or hydroxyl ions (OH^−^) and interact with other bacterial communities, and the decrease in the relative abundance may also be related to the increase in pH (Nunes da Rocha et al., 2013).

### The relationship between pH changes and RKN infection

Remarkably, we observed that RKN infection further increased the pH in weakly alkaline group 1 soil, while weakly acidic group 2 soil experienced a further decrease in pH. The results in group2 were consistent with other studies conducted in Hubei and Fujian provinces (Li et al., 2023; Lu et al., 2023). It is speculated that plants secrete organic acids and alter the microenvironment to inhibit nematode diseases and potential subsequent pathogen infections. Regarding the pH increase observed after RKN infection, it is possible that ammonium metabolism contributes to this phenomenon. When tobacco roots secrete a compound called “nodulin,” it can initiate a symbiotic relationship with *Rhizobium* (Oldroyd, 2013), promoting the growth of nitrogen-fixing bacteria and converting nitrogen from the rhizosphere soil into fertilizer, which, in turn, provides nutrients for tobacco. Furthermore, the release of nodulin could also offset the acidic substances in the rhizosphere soil, leading to a rise in pH value. In our follow-up analysis, the genus *Nitrospira* in group 1 was also found to be associated with ammonium metabolism. The association between RKN infection and changes in rhizosphere pH is not fully understood. However, our hypothesis suggests that plants may preferentially adopt strategies that best suit their self-protection based on the original physicochemical properties of the rhizosphere soil, such as Ca and P concentrations, to mitigate or suppress the adverse effects of RKN infection.

### Factors influencing the relationship between RKN infection and rhizosphere microflora changes

The interactions between microbiomes in the root rhizosphere soil play essential roles in maintaining a stable microbial community and plant health (Chen et al., 2022). Co-occurrence patterns and microbial networks are effective ways to identify key and important species. In our study, we constructed microbial networks and microbe–soil environmental factor networks to depict the complex interactions. The results showed that there were much more complex microbial interactions in group 1 than in group 2 (Figure 5). In group1, the genus *Ferruginibacter*, classified as phylum Proteobacteria, increased in relative abundance after RKN infection and positively correlated with *Streptomyces* and *Kribbella*, both belonging to phylum Actinobacteria. It also shared a negative correlation with genera *Haliangium*, *Paludibaculum,* and *Nitrospira*. Another example is the increase in relative abundance of the genus *Ramlibacter* (p__Proteobacteria) in the diseased root soil, which is positively related to *Arthrobacter* (p__Actinobacteriota) and *Massilia* (p__Proteobacteria) and highly negatively correlated with *norank_f__Pirellulaceae*, *norank_f__Pirellulaceae*, and *Methyloceanibacter* (p__Proteobacteria; Supplementary Table S7). These findings illustrate that these bacteria may be a keystone species in microbial community interactions and play an important role in defense against nematode infection and nutrient uptake. Many species of bacteria in group 2, such as *UTCFX1*, *Tundrisphaera*, *Acidothermus*, and *Actinospica*, changed in abundance due to the decreased relative abundance of the genus *Pirellula* (p__Planctomycetota). *Pirellula* has been reported to perform photosynthesis and is widely found in the ocean. Some reports have shown that *Pirellula* may be related to energy supply and substrate conversion in soil (Rabus et al., 2002). The results of correlation networks showed that six environmental factors, namely, pH, Ca, P, Mg, Fe, and Na, may be closely related to changes in the microbial flora (Figure 4), with pH being the most important factor. In group 2, only a few microorganisms were just correlated with Ca or Mg but were more closely correlated with pH changes at the same time (Figure 6). It indicated that bacterial communities under weakly acidic conditions may be more susceptible to pH changes, which is consistent with the significant influence of soil pH on microbial flora reported in other studies (Li et al., 2021; Tian et al., 2021).

Two interesting findings emerged from this study. First, there were still a large number of species with unclear taxa, many of which could not even be confirmed at the order or class level. Many unclassified species had significantly increased or decreased relative abundance (Figure 2). Additionally, numerous unclassified species were significantly correlated with multiple environmental factors (Figure 6). These results indicated that the presence of a large number of function-unknown species in the tobacco rhizosphere soil warrant further study. Second, there were many genera with abundances less than 1%, accounting for approximately 30% of the total, which was even higher than the abundance of the most abundant species (Supplementary Figure S1). This indicated that the composition of tobacco rhizosphere microorganisms was quite complex. Some reports have suggested that low-abundance species may also play specific biological roles in rhizosphere microecology (Ding et al., 2015; Leff et al., 2015; Jousset et al., 2017). We included the top 400 species in terms of relative abundance to construct different networks and found some key bacteria in low relative abundance, such as genera *Solirubrobacter* and *Phaselicystis*, although the underlying mechanism is still unknown.

## Conclusion

In this study, we collected samples from 28 tobacco-growing areas in 10 provinces in China and examined the composition of rhizosphere bacteria in healthy and infected plants in detail. Significant differences were found in the microbial composition, especially the core bacteria at the class and genus levels, between the four Northern provinces and the other six provinces, indicating distinct regional characteristics. RKN-infected tobacco exhibited a richer and more diverse rhizosphere soil bacterial community compared with healthy tobacco in most growing areas. Environmental factors such as pH, calcium (Ca), magnesium (Mg), phosphorus (P), iron (Fe), and sodium (Na) may play key roles in changing the population composition of rhizosphere microorganisms during RKN infection. Three genera were identified as potential biocontrol or plant growth-promoting bacteria for tobacco. This research provides valuable reference data for RKN disease in different tobacco-growing areas and helps explore new and effective biological control methods.

## Data availability statement

The data presented in this study is deposited in the NCBI database under BioProject accession number PRJNA971867 (https://www.ncbi.nlm.nih.gov/bioproject/PRJNA971867).

## Author contributions

YC: Conceptualization, Supervision, Writing – original draft, Funding acquisition. NL: Data curation, Investigation, Visualization, Writing – original draft. DY: Investigation, Writing – original draft. MM: Investigation, Resources, Writing – review & editing. K-QZ: Supervision, Writing – review & editing. CL: Investigation, Resources, Writing – original draft. SS: Investigation, Resources, Writing – review & editing.

## Supplementary material

### Supplementary TABLE 1

Basic information of each sample.

### Supplementary TABLE 2

Sequence results of each sample.

### Supplementary TABLE 3

Physical and chemical parameters statistics of each sample.

### Supplementary TABLE 4

Annotation at genus and phylum levels of all samples.

### Supplementary TABLE 5

Shared and unique species at the genus and phylum levels between soil from healthy and RKN infected plants.

### Supplementary FIGURE 1

Composition of microorganisms at the genus level in healthy and RKN-infected tobacco from different provinces.

### Supplementary FIGURE 2

Changes in richness and diversity of rhizosphere soil microorganisms of healthy and RKN-infected tobacco on OTU level in Guizhou and Chongqing areas.

### Supplementary FIGURE 3

Correlation analysis between the bacterial community structure at genus level and soil environmental factors of all samples without RKN disease.
