# Supplementary figures and images for: Root-knot nematode infections and soil characteristics significantly affected microbial community composition and assembly of tobacco soil microbiota: a large-scale comparison in tobacco-growing areas
Source: Front Microbiol. 2023 Dec 1;14:1282609. doi: 10.3389/fmicb.2023.1282609 (PMC10722292; doi:10.3389/fmicb.2023.1282609)

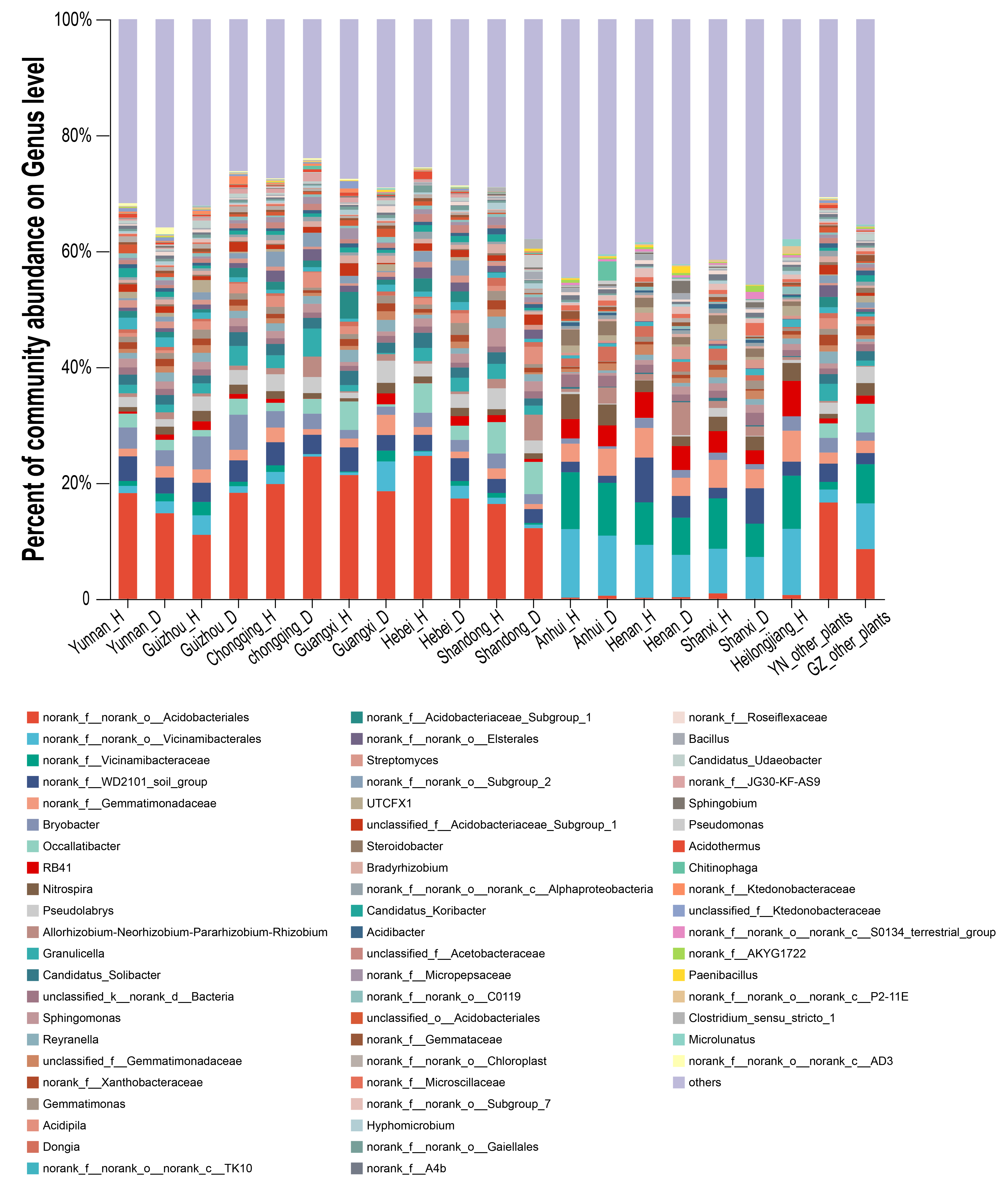

Supplement: Supplementary file 7 [file Image_1.tif]

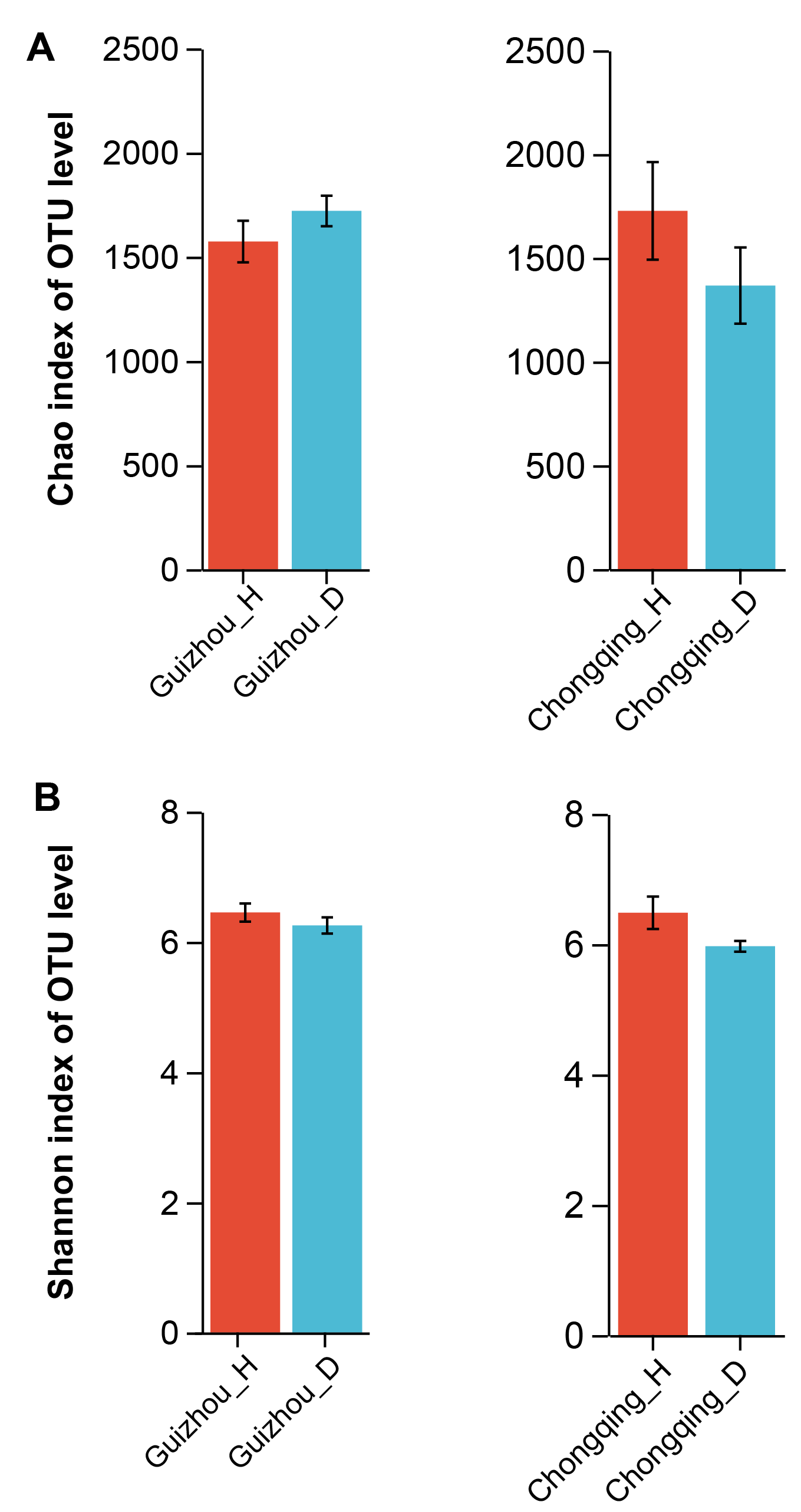

Supplement: Supplementary file 8 [file Image_2.tif]

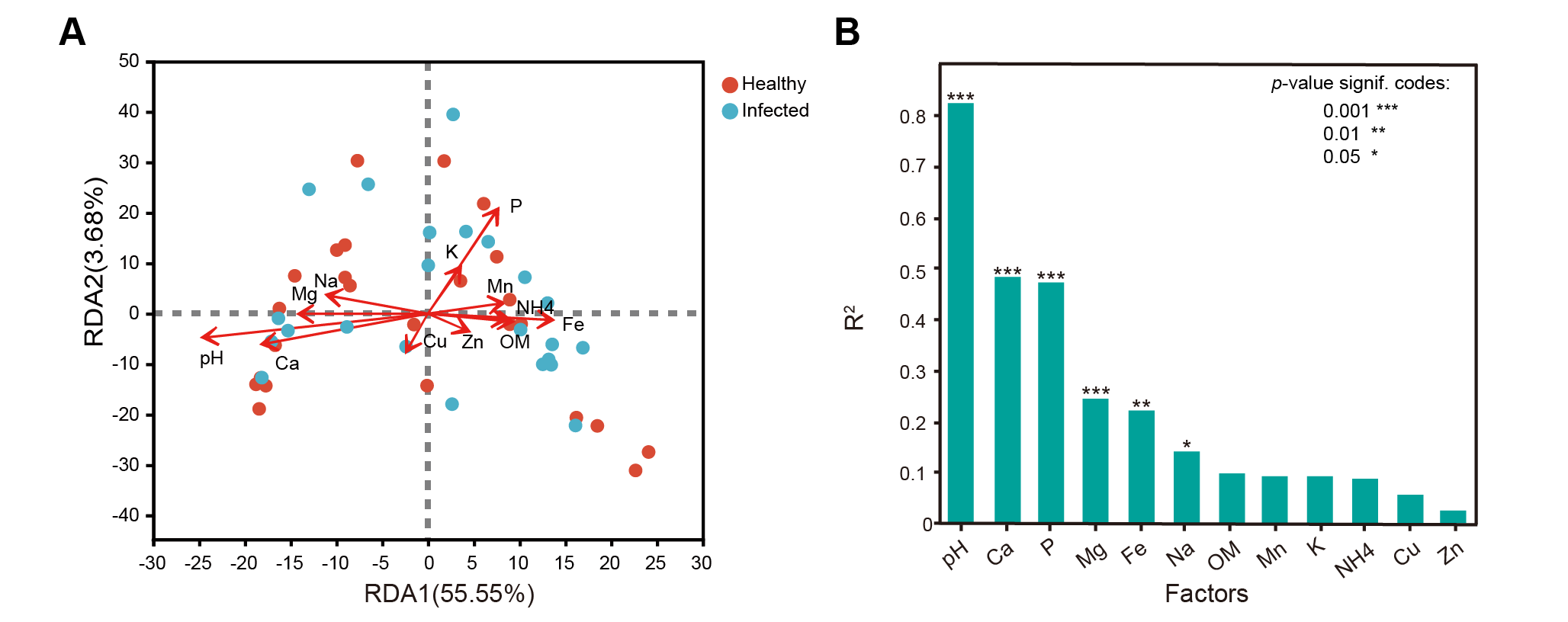

Supplement: Supplementary file 9 [file Image_3.tif]
